# Art‐omics: multi‐omics meet archaeology and art conservation

**DOI:** 10.1111/1751-7915.13480

**Published:** 2019-08-27

**Authors:** Cristina Vilanova, Manuel Porcar

**Affiliations:** ^1^ Darwin Bioprospecting Excellence SL Catedrático Agustín Escardino, 9 46980 Paterna Spain; ^2^ Institute for Integrative Systems Biology I^2^SysBio (Universitat de València‐CSIC) Parc Científic de la Universitat de València C. Catedràtic José Beltrán 2 46980 Paterna Spain

## Abstract

Multi‐omics can informally be described as the combined use of high‐throughput techniques allowing the characterization of complete microbial communities by the sequencing/identification of total pools of biomolecules including DNA, proteins or metabolites. These techniques have allowed an unprecedented level of knowledge on complex microbial ecosystems, which is having key implications in land and marine ecology, industrial biotechnology or biomedicine. Multi‐omics have recently been applied to artistic or archaeological objects, with the goal of either contributing to shedding light on the original context of the pieces and/or to inform conservation approaches. In this minireview, we discuss the application of ‐omic techniques to the study of prehistoric artworks and ancient man‐made objects in three main technical blocks: metagenomics, proteomics and metabolomics. In particular, we will focus on how proteomics and metabolomics can provide paradigm‐breaking results by unambiguously identifying peptides associated with a given, palaeo‐cultural context; and we will discuss how metagenomics can be central for the identification of the microbial keyplayers on artworks surfaces, whose conservation can then be approached by a range of techniques, including using selected microorganisms as ‘probiotics’ because of their direct or indirect effect in the stabilization and preservation of valuable art objects.

## Graphical abstract

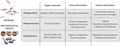

## Biomolecules and archaeology: from targeted analysis to high‐throughput, untargeted approaches

Archaeology is a discipline under constant evolution, in which numerous methodologies are typically combined in order to get more knowledge about past civilizations, including their lifestyles, their relationships with the environment, their traditions and their social organization. Very often, the same combination of techniques and procedures are applied with the purpose of preserving the cultural heritage, which is a great challenge for our own society. Archaeology is thus a deeply interdisciplinary field. The choice of the technique or techniques to be applied in archaeological research depends on the goal of the study, which can focus on the chronological determination for some event in the past (David *et al*., 2013; Sauvet, 2015; Bonneau *et al*., 2017), identifying the identity of biological traces (Kamp *et al*., 1999; Kamp, 2001; Crown *et al*., 2009) or analyzing the composition of a particular object (Reiche *et al*., 2011; Brandt *et al*., 2014). Several factors such as the type of material under study (i.e. rock art, man‐made objects and hearths), the requirements of the technique (i.e. invasive vs non‐invasive) and the versatility of the generated data strongly determine the experimental design and the methodology chosen for archaeological research.

In particular, physico‐chemical analysis are the main group of methodologies enabling the investigation of the origin of archaeological objects and the raw materials used for their construction, the description of the elaboration processes of human‐made objects or artworks, and the follow‐up of conservation and restoration efforts. Three main types of physico‐chemical techniques have traditionally been applied in this field (Neff, 1993): (i) thermal analysis, (ii) mineralogical‐crystallographic analysis and (iii) techniques for the identification and quantification of chemical elements or compounds. The latter group includes, among others, a range of physico‐chemical techniques targeting specific biomolecules such as DNA, proteins and other metabolites.

Even though the vast majority of biomolecular techniques are invasive and require the destruction of the sample, they have greatly expanded among the archaeological community in the last decades due to the valuable information they are able to produce. In origin, these techniques were designed to target particular biomolecules with a high specificity. For instance, specific PCR (Polymerase Chain Reaction) protocols were developed to analyze hypervariable regions of human DNA (Harder *et al*., 2012; Olasz *et al*., 2019), and a range of ELISA (Enzyme‐Linked ImmunoSorbent Assay) protocols were designed for the detection of particularly relevant proteins such as albumin (Cattaneo *et al*., 1994), animal keratin (Wang *et al*., 2018) or silk fibroin (Liu *et al*., 2015) in ancient textiles. However, the development of the so‐called omic techniques (genomics, proteomics and metabolomics), able to perform a complete analysis of the total pool of DNA (genome), proteins (proteome) and other metabolites (metabolomics) in a single experiment (Jansson and Baker, 2016), has dramatically changed the nature of the biomolecular studies applied in the field of archaeology. The two main advantages of ‐omic technologies for archaeological research are their untargeted nature and the high‐throughput generation of data, since they are able to simultaneously yield information on a broad repertoire of biomolecules present in a sample. Hence, with the same amount of sample used in traditional biomolecular analysis to obtain information on a given molecule, omic technologies are able to generate an exhaustive dataset on thousands of molecules. In the following sections, the application of proteomic, metabolomic and metagenomic techniques in archaeological research (especially in the study of ancient art and objects) is briefly reviewed.

## Application of proteomic and metabolomic techniques to the study of artwork and ancient man‐made objects

Multi‐omics have recently arisen as strong, alternative approaches for chronologic and conservation purposes of artistic and/or prehistoric objects. The main goal of proteomics on ancient art paintings has been the identification of the proteinaceous binders used for the preparation of the pigments. The two main approaches for this type of studies consist of (i) untargeted LC–MS/MS (liquid chromatography coupled to tandem mass spectrometry), aimed at identifying a broad representation of the peptides present in the sample; and (ii) MRM (multiple reaction monitoring), targeting particular peptides which are known markers for a range of putative binders. This is the case of casein (an obvious protein marker for milk), ovoalbumin (egg) or vitellogenin (yolk) peptides. These techniques have been applied to the analysis of ancient protein binders in a range of prehistoric art elements. The oldest sample analyzed by proteomics is an ochre‐painted stone flake from a 49 000‐year‐old layer of Sibudu (South Africa), in which casein from a wild bovid was detected as the main protein binder (Villa *et al*., 2015). Other sound cases include the proteomic analysis of the first historical overpaintings of the lost Giant Buddhas of Bāmiyān (Afghanistan), where a mixture of milk from different mammals was pointed out as the most likely binder (Lluveras‐Tenorio *et al*., 2017), and a set of rock art paintings from the Mediterranean basin (Valltorta, Spain), in which bovid casein traces were also detected (Roldán *et al*., 2018). The latter case is an example of how proteomic techniques can shed light not only on the composition of the binders, but also on art dating, since the detection of milk from wild or farmed animals has direct implications in the putative lifestyle of the authors of the art elements (i.e. early stockbreeding communities). Indeed, it has to be stressed that the Levantine rock art lacks a consensus dating and it is estimated to span over a culturally crucial period between, approximately 8000 and 3000 years BC. This period saw a transition from hunter‐gatherer economic systems to a Neolithic society (http://whc.unesco.org/en/list/874). The paintings include animals that could be used as cattle, and the identification of milk on the paintings would thus further support this conclusion (Roldán *et al*., 2018).

Out of the art framework, proteomics has been also essential to unveil the composition of food remains found in ancient ceramic vessels, yielding valuable evidence of the lifestyle and traditions of prehistoric societies. A range of dietary foodstuffs, including dairy, cereal grains, legumes and non‐dairy animal proteins were detected in ceramic sherds from the early farming site of Çatalhöyük, in central Anatolia (Hendy *et al*., 2018). Animal milk proteins were also detected in the ancient Subeixi region, the furthest eastern location of prehistoric milking in the Old World (Hong *et al*., 2012), and evidence for the early exploitation of aquatic resources was obtained in pottery from inland prehistorical localities in Brandenburg (Germany), where processed fish caviar meal traces were detected through proteomics (Shevchenko *et al*., 2018).

Finally, the proteomic analysis of other ancient objects such as wooden staffs (Rao *et al*., 2015), baskets (Solazzo *et al*., 2016) and textiles (Gong *et al*., 2016) has enabled the detection of bovine and fish tissues used as glues or coatings and the early use of silk for textile elaboration. Albeit controversial, the direct proteomic analysis of archaeological soils has been proposed as a way to detect ancient hairs, feedstock remains and other traces during the sampling of archaeological sites (Oonk *et al*., 2012).

On the other hand, different biomolecules such as lipids and secondary metabolites have been analyzed through metabolomics, by using GC–MS (gas chromatography coupled to mass spectrometry) to investigate the composition of art elements and prehistoric objects beyond their protein binders. This is the case of paint microsamples from different artworks (a painted hull of a shipwreck, an easel painting and a painted Etrurian sarcophagus), where linseed oil, beeswax and Pinaceae resin were identified as part of the paint mixture (Andreotti *et al*., 1978). However, the most informative studies based on GC–MS shed light on the contents of ancient pottery or other archaeological elements. The oldest objects analyzed with this approach are indeed pottery elements from Xianrendong Cave (China), dated about 20 000–19 000 years BP, in which the lipid composition (rich in markers dominated by medium‐ and long‐chain saturated and monounsaturated fatty acids and isoprenoid fatty acids) suggested the use and storage of high‐trophic‐level aquatic food (Craig *et al*., 2013). Similar results were later obtained in a study comprising samples from a wide chronological spectrum (9000‐years sequence) from the Jōmon site of Torihama, in Western Japan (Lucquin *et al*., 2016). The first direct evidence for the exploitation of palm fruit in antiquity and the use of pottery vessels in its processing was obtained by means of the same technique on samples from Qasr Ibrim (Egypt) (Copley *et al*., 2001).

GC–MS lipid analysis has also contributed to archaeological evidences in the analysis of different sites within hearths. Despite the degradation suffered by lipids as a consequence of fire or other catastrophic events, the information obtained from key fatty acid ratios has helped to distinguish between plant and animal lipids, and in some cases can even help to identify specific plant and animal taxa (Kedrowski *et al*., 2009).

## The particular case of metagenomics: conservation and art probiotics

The microbial composition of ancient, artificial objects or artworks has been studied with some detail with worldwide examples, although many of those were limited because of the use of only culture‐dependent techniques. For example, a collection of actinomycetes strains from mural Egyptian paintings has been reported (Elhagrassy, 2018). These isolates proved able to modify the colour of the original pigments while, paradoxically, were also able to biocontrol potential microbial degraders (Elhagrassy, 2018). A painting in Herculanum, Italy, was also studied through culture techniques that allowed the identification of heterotrophic bacteria and fungi (Pepe *et al*., 2011). Culture‐depending techniques can yield a moderately diverse set of microorganisms. Indeed, the isolation of novel species from ancient artworks is not rare, and examples include the *Myroides* new species isolated from prehistoric paintings in Bulgaria (Tomova *et al*., 2013); several *Rubrobacter* strains from deteriorated ancient monuments (Laiz *et al*., 2009); and a new *Paenibacillus* and several new *Arthrobacter* species from a Roman tomb in Spain (Heyrman *et al*., 2005; Smerda *et al*., 2006). That said, Next Generation Sequencing (NGS) techniques can obviously yield a much broader view of the actual biological diversity of the microbial communities associated with man‐made ancient objects.

Duan *et al*. (2017) reported a culture‐independent Illumina MiSeq sequencing characterization of the microbial composition of the Maijishan Grottoes in a cave in China, a World Cultural Heritage site listed by UNESCO, by sampling paintings carried out during the Northern Zhou Dynasty (557‐581AD). A rich bacterial diversity including the bacterial groups Actinobacteria, Acidobacteria, Bacteroidetes, Cyanobacteria, Chloroflexi, Firmicutes, Proteobacteria and Verrucomicrobia was unveiled, while fungi exhibited a lower diversity, consisting of Ascomycota, Basidiomycota and Chytridiomycota. Some of these microorganisms, particularly the *Pseudonocardia* genus, have been reported to be associated with biodeterioration of art pieces or other culturally relevant objects, such as Etruscan paintings in Tuscany suffering from a heavy microbial colonization (Diaz‐Herraiz *et al*., 2013). In Nara, Japan, Sugiyama *et al*. (2017) used a combination of culture‐dependent and culture‐independent techniques to study some of the 1300‐year‐old polychrome mural paintings of the historical sites of Takamatsuzuka and Kitora Tumulus, which resulted in the identification of a new bacterial species of the *Stenotrophomonas* genus (Handa *et al*., 2016). Interestingly, severe outbreaks of microbial contaminants were reported, involving genera such as *Doratomyces* sp. and *Streptomyces* sp. Such outbreaks even required dramatic corrective actions such as UV light treatments, with limited success in controlling some of the microbial keyplayers in degradation (Sugiyama *et al*., 2017) .

One of the first culture‐independent studies performed on rock paintings describe an important pool of novel species in Altamira, Spain (Schabereiter‐Gurtner *et al*., 2002). The authors concluded that the high number of clones that were most closely related to environmental 16S rDNA clones suggests a wide set of unknown bacteria in the cave. In this same location, other researchers further reported that the metabolically active microbial community, dominated by proteobacteria, is only a small fraction of the total one, which implies that microorganisms with undetectable activity are paradoxically a potential risk for the conservation of ancient artworks since environmental variations can trigger their flourishing (Portillo *et al*., 2008).

The current development of NGS techniques applied to art conservation implies that an accurate knowledge of the microbial composition of paintings, buildings or sculptures is accessible with relatively fast and easy techniques. Beyond taxonomic characterization and the eventual description of new taxa, a major goal of metagenomics is the artificial modification of the microbiome, often with the objective of improving the pieces’ conservation. For example, a recent report describing the microbiome of a 17th century baroque easel demonstrated the potential of *Bacillus spp*. to control fungal populations associated with poor conservation and deterioration of the pictural layer (Caselli *et al*., 2018). The use of microorganisms in restoration of painting and other artworks, often defined as ‘biocleaning’ has been shown to be less hazardous than traditional, mechanical or chemical techniques, as well as to be non‐toxic for both restorers and the environment. One example of a bacterium used in biocleaning is *Pseudomonas stutzeri* strain 5190, which can use a wide range of carbon and nitrogen sources and is very tolerant to several stressing abiotic factors found on the painting surface (Bosch‐Roig *et al*., 2016). Paradoxically, these strategies imply the use of culture techniques, which are still imperative to carry out in controlled environments the confrontation tests between target and ‘probiotic’ microorganisms. This is, in our view, a very good example of both the power and limitations of ‐omic techniques, as we have previously highlighted (Vilanova and Porcar, 2016): such techniques are a priceless source of biological information, but yet require cultivation when it comes to translate massive genomic information into practical (i.e. conservation) actions.

## Beware of degradation and modern contaminants: current limitations of ‐omic studies in archaeological research

**Figure 1:**
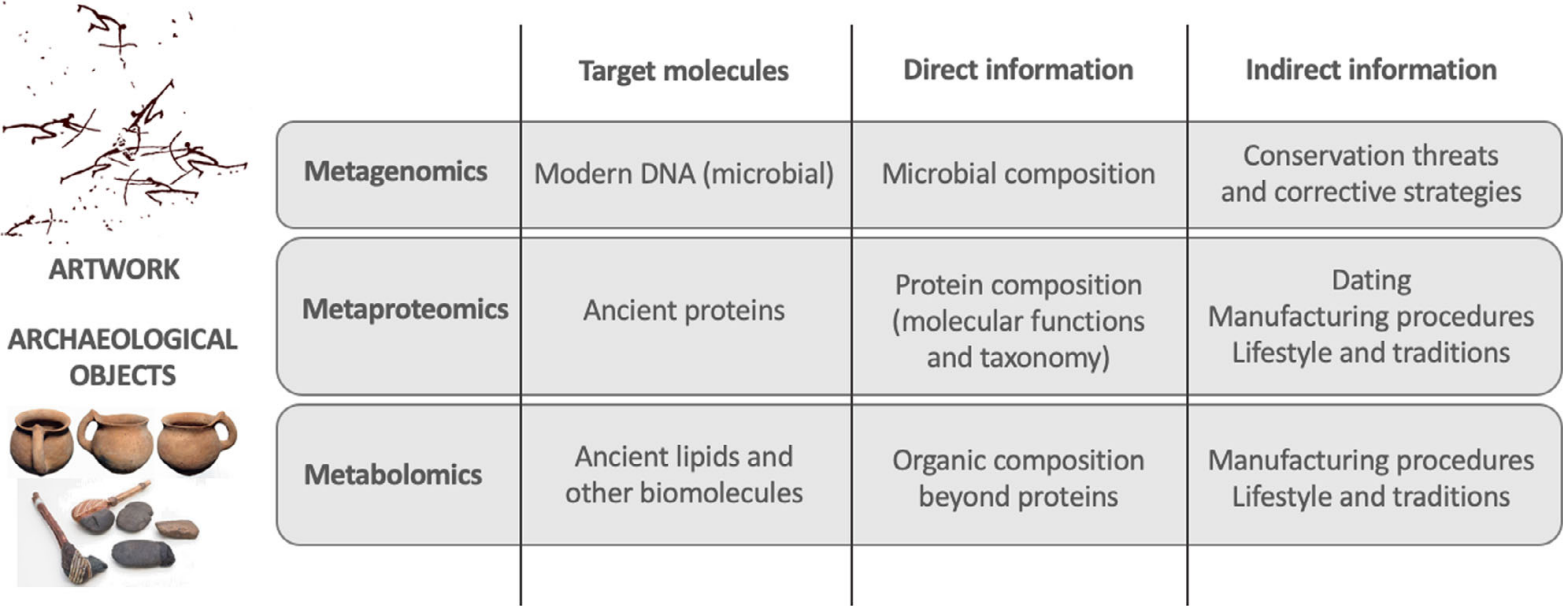
Summary of ‐omic approaches applied to the study of artwork and archaeological objects and expected evidences depending on the biomolecules under study.

Art and archaeological objects are always precious materials that are central to contribute to the understanding of our cultural and biological history. Multi‐omics (Fig. 1) have arisen as a new range of techniques that have proven helpful to either indirectly calculating the chronology or the cultural context of the pieces, as well as to provide relevant information on potential microbial degraders and stabilizers of the objects (typically, but not restricted to, paintings). That said, there are important limitations of the assays linked to the degradation of biomolecules in time and the contamination of the samples with modern biomolecules.

On the degradation of biomolecules, it is well known that DNA can survive up to hundreds of thousands of years under certain circumstances, but in practical circumstances and under already human‐contaminated and/or warm climates, the determination of the palaeo‐metagenomic composition of a piece may be impossible. Therefore, and in normal conditions, a metagenomic DNA analysis will provide insights on the actual microbial community on the object, not the original one. By contrast, both proteins and lipids are less sensitive to degradation (Evershed *et al*., 1990; Cappellini *et al*., 2012), and therefore are suitable markers for determining the ancestral composition of an object/sample. Nevertheless, it has to be noted that microbial degradation can play a significant role in lipid degradation in archaeological samples (Malainey *et al*., 1999; Evershed *et al*., 2002).

On the other side, and especially in the case of proteomic studies, contamination of human origin is almost always present in the samples and can be due to a poor sub‐recent preservation frame (i.e. prehistoric paintings vandalized during the last years/decades) and/or to inadequate handling of the samples (objects handled without gloves, for example). That is why specific strategies for the filtering of modern contaminants have been set in place, being the analysis of glutamine deamidation rates (Wilson *et al*., 2012; Solazzo *et al*., 2014) the most reliable method to distinguish ancient peptides (with high deamidation levels) from contemporary ones (showing a low rate of deamidation). However, a range of factors different from age (i.e. preservation conditions, protein primary sequence and tridimensional structure) are known to influence glutamine deamidation (Schroeter and Cleland, 2016), so the direct correlation between deamidation rates and protein antiquity cannot be taken for granted in all cases. This suggests the need of combining such analytical methodologies with a robust sampling strategy, including a range of control samples, which may help to detect modern contaminant proteins.

Provided that suitable controls are set, multi‐omics will certainly consolidate as powerful tools to contribute to shed light on our past as well as to preserve modern and ancient work pieces. We anticipate that the current progress in proteomic analysis in the frame of human palaeontology will be linked to a parallel explosion of protein‐ and maybe metalobite‐based studies on man‐made objects, which may translate into a revolutionary *biologization* of the way we approach our past.

## Authors’ contribution

Both authors conceived, wrote, read and approved the final manuscript.

## Conflict of interest

None declared.
